# Urine Biochemistry in the Early Postoperative Period after Cardiac Surgery: Role in Acute Kidney Injury Monitoring

**DOI:** 10.1155/2013/103450

**Published:** 2013-07-28

**Authors:** Alexandre Toledo Maciel, Daniel Vitório

**Affiliations:** Intensimed Research Group, Adult Intensive Care Unit, Hospital São Camilo, Pompéia Avenue, 1178 Pompéia, 05022-001 São Paulo, SP, Brazil

## Abstract

We have recently suggested that sequential urine electrolyte measurement in critically ill patients may be useful in monitoring kidney function. Cardiac surgery is one of the leading causes of acute kidney injury (AKI) in the intensive care unit (ICU). In this paper, we describe the sequential behavior of urine electrolytes in three patients in the early (first 60 hours) postoperative period after cardiac surgery according to AKI status: no AKI, transient AKI, and persistent AKI. We have found that the patient with no AKI had stable and high concentrations of sodium (NaU) and chloride (ClU) in sequential spot samples of urine. AKI development was characterized in the other two patients by decreases in NaU and ClU, which have started early after ICU admission. Transient AKI was marked by also transient and less severe decreases in NaU and ClU. Persistent AKI was marked by the less favorable clinical course with abrupt and prolonged declines in NaU and ClU values. These electrolytes in urine had a behavior like a “mirror image” in comparison with that of serum creatinine. We suggest that sequential urine electrolytes are useful in monitoring acute kidney injury development in the early postoperative period after cardiac surgery.

## 1. Introduction

Acute kidney injury (AKI) is frequent among patients undergoing cardiac surgery [1, 2]. It seems to be an independent risk factor for increased intensive care and hospital mortality [3]. Serum creatinine level and urine output are still the cornerstones for AKI diagnosis in all settings, including postoperative AKI. Urine biochemistry, although a major tool in AKI diagnosis and management in the past, is nowadays considered not useful [4] especially due to evidence showing its dissociation from renal hemodynamics [5]. However, sequential evaluation of urine electrolytes (basically, sodium, potassium, and chloride) in the course of early postoperative period has never, to our knowledge, been performed. We have recently observed that alterations in the concentration of these electrolytes measured in spot urine samples may be related to kidney function and AKI development, sometimes preceding elevations in serum creatinine [6, 7]. In this paper, we report the sequential behavior of urine electrolytes in a 60 h period in three patients after undergoing cardiac surgery. 

## 2. Case Presentation

We will briefly present the 3 cases separately. In all cases, there was no previous history of kidney disease, and the surgical procedure consisted in on-pump coronary artery bypass graft (CABG), and all patients had their serum creatinine as well as urine sodium (NaU), chloride (ClU), and potassium (KU) measured at 0 (*T*_0_), 6 (*T*_6_), 12 (*T*_12_), 24 (*T*_24_), 36 (*T*_36_), 48 (*T*_48_), and 60 (*T*_60_) hours after ICU admission. These measurements are part of a research protocol in our ICU of which these patients were the first three included. All patients were admitted in the ICU immediately after the surgery. An indwelling urinary catheter was in place during the entire observation period. AKIN creatinine-based criteria were used to define AKI [8]. Baseline creatinine was considered the creatinine value at ICU admission. Oliguria was defined as a urine output less than 0.5 mL/kg/h in a 6 h period. AKI reversal was defined as a creatinine value lower than baseline creatinine +0.3 (mg/dL). Day 1 (D1) is the day of ICU admission. 

### 2.1. Patient 1 (P1)

**Figure 1:**
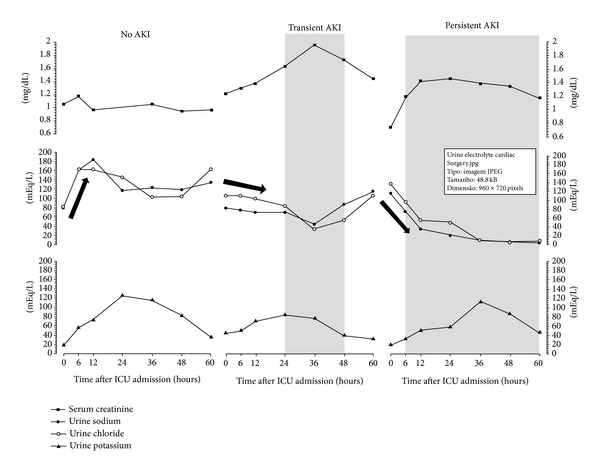
Sequential serum creatinine and urine electrolyte concentrations in the first 60 hours after intensive care unit admission of 3 patients in the postoperative period of cardiac surgery. Patient 1 did not develop creatinine-based AKI (left); patient 2 developed a transient AKI (middle), and patient 3 developed a persistent AKI (right). AKI: acute kidney injury.

P1 was a 67-year-old female with a past medical history of hypertension, dyslipidemia, and hypothyroidism in which CABG was electively indicated due to stable angina. Three coronary bypasses were performed in 67 minutes of cardiopulmonary bypass (CPB) and 51 minutes of aortic clamping (AC). No vasopressors were needed neither intraoperatively nor postoperatively. She was extubated 5 hours after ICU admission. Creatinine remained stable around 1 mg/dL in all measurements (unfortunately, creatinine value at *T*_24_ was missed) (Figure 1). Oliguria occurred between *T*_12_ and *T*_24_, for which furosemide was administered. It was also administered between *T*_48_ and *T*_60_. NaU increased progressively from *T*_0_ to *T*_12_, decreasing a little between *T*_12_ and *T*_24_, remaining stable after that, and increasing again at *T*_60_. ClU behavior was similar to that of NaU (Figure 1). KU increased progressively until *T*_24_, decreasing progressively after that. The rest of ICU stay was unremarkable, and P1 was discharged at D4.

### 2.2. Patient 2 (P2)

P2 was a 74-year-old female with a past medical history of hypertension, diabetes mellitus, dyslipidemia, and hypothyroidism in which CABG was indicated electively a few days after an acute coronary syndrome. The patient was stable before the procedure. Three coronary bypasses were performed in 105 minutes of CPB and 78 minutes of AC. No vasopressors were needed intraoperatively. She was extubated 9 hours after ICU admission. Norepinephrine infusion was initiated due to hypotension between *T*_12_ and *T*_24_, remaining in low doses until *T*_60_. No diuretics were needed between *T*_0_ and *T*_60_, although oliguria also occurred between *T*_24_ and *T*_36_. Creatinine increased progressively until it reached AKIN stage 1 at *T*_24_ (Figure 1). Peak creatinine was reached at *T*_36_, and AKI reversal occurred at *T*_60_. NaU and ClU had small decreases between *T*_0_ and *T*_24_ and reached their lowest values at *T*_36_. KU increased progressively until *T*_24_, decreasing progressively until *T*_60_. Norepinephrine infusion was stopped at D4, and P2 was discharged at D5. 

### 2.3. Patient 3 (P3)

P3 was a 71-year-old female with a past medical history of hypertension, diabetes mellitus, chronic pulmonary obstructive disease, and ischemic stroke in which CABG was also indicated electively a few days after an acute coronary syndrome. The patient was stable before the procedure. Four coronary bypasses were performed in 85 minutes of CPB and 64 minutes of AC. Norepinephrine was needed both intraoperatively and postoperatively (including doses above 0.5 mcg/kg/min), during the entire observation period (*T*_0_–*T*_60_). She was extubated 5 hours after ICU admission. Furosemide and bicarbonate were infused between *T*_6_ and *T*_12_ due to metabolic acidosis and oliguria. Furosemide was also repeated frequently between *T*_12_ and *T*_60_ due to oliguria and pulmonary edema. Creatinine increased fast, and AKIN stage 1 was present at *T*_6_ (Figure 1). Peak creatinine was reached at *T*_24_, decreasing slowly in the subsequent measurements but without reaching AKI reversal criterion until *T*_60_. Both NaU and ClU decreased fast and to very low levels (<10 mEq/L) at *T*_48_–*T*_60_ (Figure 1). KU increased progressively reaching its peak at *T*_36_. Norepinephrine infusion was stopped at D5, and P3 was discharged at D6.

## 3. Discussion

The 3 reported cases described above show 3 distinct evolutions during and after CABG surgery. P1 had a more benign course with no need of vasopressors at anytime and no significant elevations in creatinine. Urine biochemistry was marked by high values of NaU and ClU at all times, increasing until *T*_12_ followed by a small fall between *T*_12_ and *T*_24_, coinciding with oliguria and high KU. Unfortunately, we cannot exclude an increase in creatinine at *T*_24_, but, even if it has happened, it decreased back to normal values in 12 hours. P2 had a worse clinical course than P1, characterized by longer periods of CPB and AC and the need of vasopressors in low doses in the postoperative period. Creatinine has gradually and transitorily increased, which was simultaneous with gradual but also transitory decreases in NaU and ClU, both reaching lower values than those reached by P1 (Figure 1). P3 had the worst clinical course characterized by prolonged use of vasopressors in high doses including the intraoperative period. An earlier and more persistent AKI developed, which was accompanied by early and abrupt decreases in NaU and ClU values until very low levels, which remained low during the entire AKI course. This may reflect the severity of the disease and might be a sign of microcirculatory impairment in the kidneys in association with activation of sympathetic and renin-angiotensin-aldosterone systems. Curiously, KU had a very similar behavior in all patients; it progressively increased reaching a peak between *T*_24_ and *T*_36_, decreasing thereafter. All these data suggest that sequential urine electrolyte measurement in the early postoperative period has standardized behaviors according to renal function: relatively preserved (oliguria without increases in creatinine) as occurred in P1 with high values of NaU and ClU, possibly due to higher glomerular filtration rate and lower microcirculatory stress. Furosemide could have contributed to increases in urine electrolytes, but P3 has used furosemide in similar doses but more frequently, and this has not increased NaU and ClU values. In fact, patients who had a more severe compromise of renal function and systemic circulation also had more significant decreases in NaU and ClU. AKI recovery was followed by increases in NaU and ClU probably due to glomerular filtration recovery and microcirculatory improvement. Very low values of NaU and ClU should not be interpreted as markers of “prerenal” impairment. This old concept seems flawed [9, 10], and low values of these electrolytes are probably markers of glomerular function impairment (regardless of total renal blood flow) together with an avid sodium retentive state in the tubules. It is noteworthy that, although many previous studies have demonstrated early increase in markers of tubular injury in postoperative AKI, including cardiac surgery [2], P3 was a case of persistent AKI that seemed to be predominantly hemodynamic, which presupposes preservation of global tubular function but does not exclude some degree of tubular injury [11]. In fact, back leak of solutes in the tubules cannot be discarded as a contributor to low NaU and ClU values in P3. 

## 4. Conclusions

Urine electrolyte concentrations in the early postoperative period after cardiac surgery are closely related to renal function and systemic hemodynamic compromise. NaU and ClU had similar behaviors, which were generally in the opposite direction of that of creatinine (as “mirror images”—see Figure 1). Decreases in NaU and ClU levels should be viewed as alert signs. An abrupt fall in NaU and ClU values is probably related to a more severe ongoing renal impairment. Transient AKI had equally transitory alterations in these electrolytes. On the other hand, persistent AKI had persistent low values of NaU and ClU. The cases reported here should be viewed as examples of the potential relevance of urine electrolyte measurement in AKI monitoring after cardiac surgery. Of particular interest is the evaluation of these electrolytes in the first 6–12 hours after surgery (black arrows in Figure 1)—NaU and ClU increase in P1 suggested preserved renal function, and decrease in P2 and especially P3 suggested some degree of renal impairment. All of these findings must be tested in a large scale as well as in other scenarios such as noncardiac postoperative period and sepsis.

## Abbreviations

AKI: Acute kidney injury
CABG: Coronary artery bypass graft
CPB: Cardiopulmonary bypass
AC: Aortic clamping
P1, P2, P3: Patients 1, 2, and 3, respectively
NaU: Spot urine sodium
ClU: Spot urine chloride
KU: Spot urine potassium.
